# Whole-genome sequencing identifies homozygous *BRCA2* deletion guiding treatment in dedifferentiated prostate cancer

**DOI:** 10.1101/mcs.a001362

**Published:** 2017-05

**Authors:** Karin Purshouse, Anna Schuh, Benjamin P. Fairfax, Sam Knight, Pavlos Antoniou, Helene Dreau, Niko Popitsch, Kevin Gatter, Ian Roberts, Lisa Browning, Zoe Traill, David Kerr, Clare Verrill, Mark Tuthill, Jenny C. Taylor, Andrew Protheroe

**Affiliations:** 1 Oxford Cancer and Haematology Centre, Churchill Hospital, Headington, Oxford OX3 7LE, United Kingdom;; 2 Oxford National Institute for Health Research, Biomedical Research Centre/NHS Translational Diagnostics Centre, The Joint Research Office, The Churchill Hospital, Headington, Oxford OX3 7LE, United Kingdom;; 3 Wellcome Trust Centre for Human Genetics, University of Oxford, Oxford OX3 7BN, United Kingdom;; 4 Nuffield Division of Clinical Laboratory Sciences, Radcliffe Department of Medicine, University of Oxford, John Radcliffe Hospital, Oxford OX3 9DU, United Kingdom;; 5 Molecular Oncology and Haematology Unit, Weatherall Institute of Molecular Medicine, John Radcliffe Hospital, Oxford OX3 9DS, United Kingdom;; 6 Department of Cellular Pathology, John Radcliffe Hospital, Oxford University Hospitals NHS Foundation Trust, Oxford OX3 9DU, United Kingdom;; 7 Department of Radiology, John Radcliffe Hospital, Oxford University Hospitals NHS Foundation Trust, Oxford OX3 9DU, United Kingdom

**Keywords:** malignant genitourinary tract tumor, prostate cancer

## Abstract

Whole-genome sequencing (WGS) has transformed the understanding of the genetic drivers of cancer and is increasingly being used in cancer medicine to identify personalized therapies. Here we describe a case in which the application of WGS identified a tumoral *BRCA2* deletion in a patient with aggressive dedifferentiated prostate cancer that was repeat-biopsied after disease progression. This would not have been detected by standard BRCA testing, and it led to additional treatment with a maintenance poly ADP ribose polymerase (PARP) inhibitor following platinum-based chemotherapy. This case demonstrates that repeat biopsy upon disease progression and application of WGS to tumor samples has meaningful clinical utility and the potential to transform outcomes in patients with cancer.

## INTRODUCTION

The role of whole-genome sequencing (WGS) in identifying targetable mutations in cancer remains one of the greatest hopes in achieving personalized cancer therapy. Its role in patients with advanced treatment-resistant cancer by rebiopsying patients at the point of progression has not been fully explored.

Most prostate cancers are adenocarcinomas, which are sensitive to androgen-deprivation therapy. It is thought that androgen receptor–depleted neuroendocrine cells, which are few in number and normally lie quiescent, can flourish because of a cascade of molecular events, resulting in neuroendocrine dedifferentiation and androgen therapy–resistant prostate cancer (Li et al. 2013, 2016). Dedifferentiation from adenocarcinoma to small-cell carcinoma is also recognized in *EGFR*-mutant lung cancer (Sequist et al. 2011).

Mutations in DNA repair genes are an important feature of many cancers and are associated with an advanced, aggressive phenotype in prostate cancer (The Cancer Genome Atlas Research Network 2015; Mateo et al. 2015; Robinson et al. 2015). Germline *BRCA* mutations and their clinical association with breast and ovarian cancer are well-known, although there is an associated increased risk of other malignancies such as stomach and pancreatic cancer (Friedenson 2005). The increased prevalence of prostate cancer in patients with germline *BRCA1* and *BRCA2* mutations has prompted consideration of prostate cancer screening in such patients by the IMPACT screening study (Bancroft et al. 2014).

Identification of somatic *BRCA* mutations in breast and ovarian cancer has been driven by the option of poly ADP ribose polymerase (PARP) inhibitor therapy for patients with *BRCA* mutations (Hennessy et al. 2009; Winter et al. 2016). Most *BRCA* mutations identified in patients with prostate cancer are somatic, and, compared with low mutation levels in early prostate cancer, ∼15% of patients with metastatic androgen therapy–resistant prostate cancer harbor functionally relevant somatic mutations or deletions in *BRCA1* or *BRCA2* (Robinson et al. 2015).

Current recommendations regarding the role for rebiopsy in prostate cancer focus on patients for whom an active surveillance approach is being pursued, although the most recent European Association of Urology (EAU)–European Society for Radiotherapy & Oncology (ESTRO)–International Society of Geriatric Oncology (SIOG) Guidelines note the importance of biopsy at the point of biochemical relapse in prostate cancer, ideally 18–24 mo after treatment (Cornford et al. 2016). Early rebiopsy or genome sequencing either at initial biopsy or rebiopsy on disease progression do not have a standard role in management. Here we present a case report of a patient with prostate cancer in whom rebiopsy and WGS at the point of disease progression had a significant and ongoing clinical impact because of the identification of a tumoral *BRCA2* deletion not present in the initial biopsy.

## RESULTS

### Clinical Presentation and Family History

**Figure 1.**
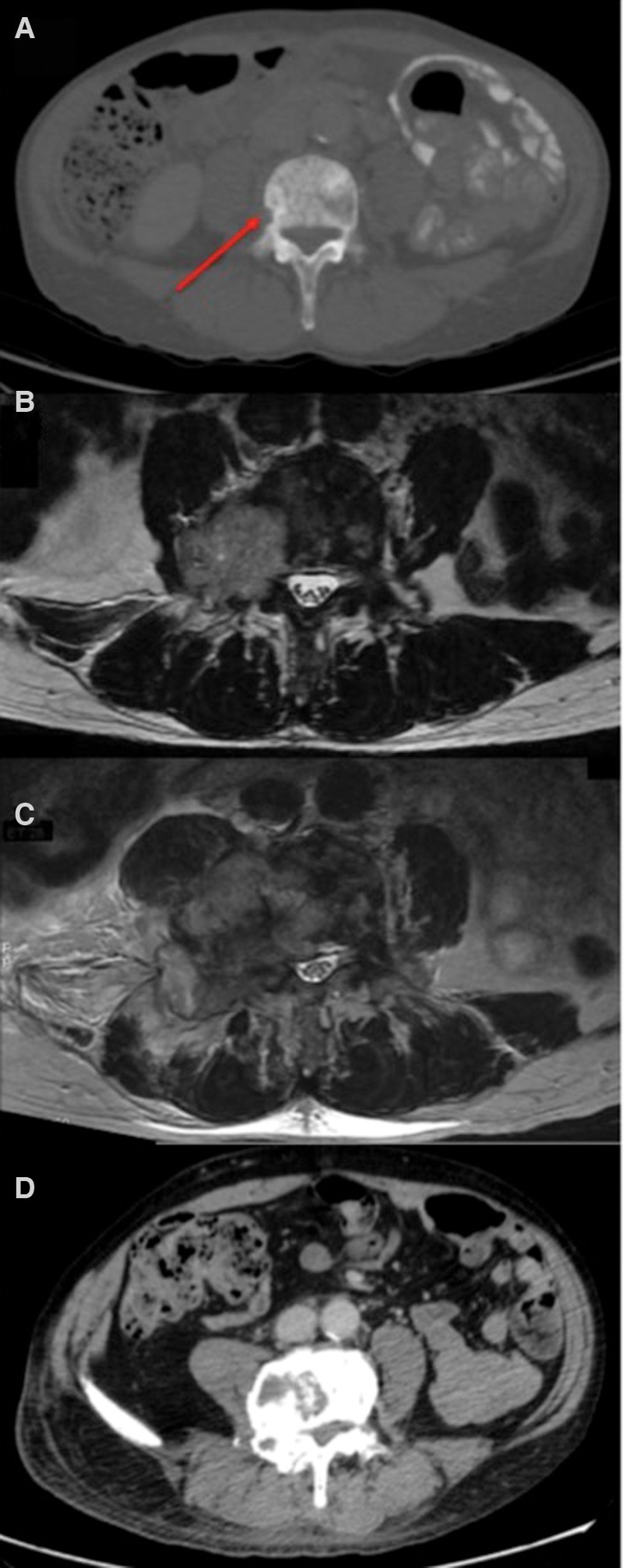
Computed tomography (CT) and magnetic resonance imaging (MRI) images demonstrating disease course. (*A*) Initial staging CT scan. Axial section of lumbar spine, soft tissue window, demonstrating mild bone erosion (arrow) indicating bone metastasis. (*B*) Image at month 2.5 (see Fig. 3 for ALP/PSA correlation)—MRI spine, axial section, demonstrating L4 lesion invading into right pedicle. (*C*) Image at month 4—MRI spine, axial section, demonstrating further increase in L4 lesion with extensive metastatic marrow infiltration. (*D*) Image at month 10.5—CT scan, bone window, demonstrating significant reduction in the lesion at L4.

**Figure 2.**
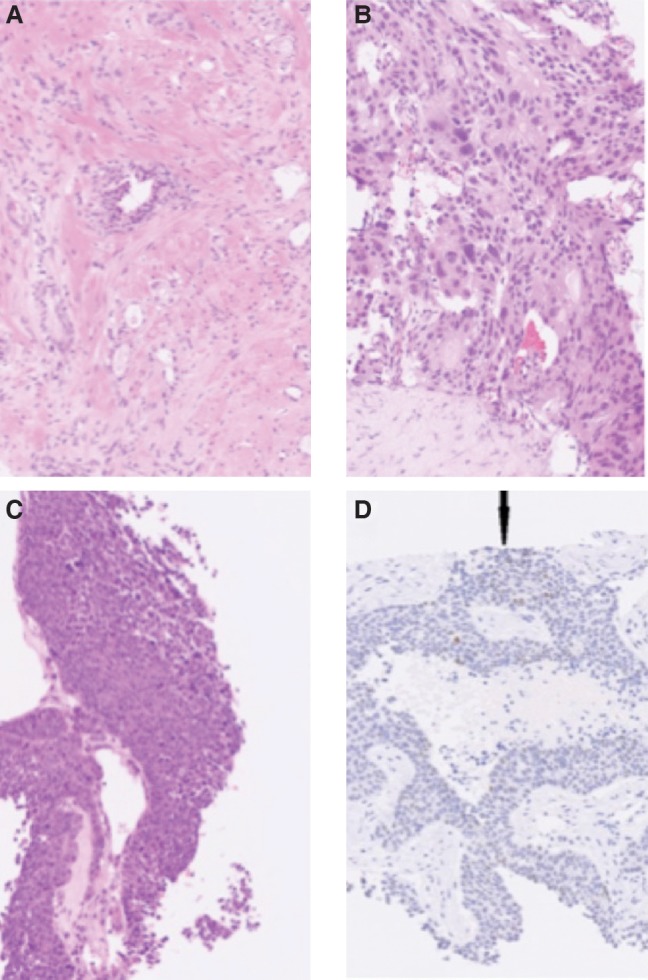
(*A*,*B*) Prostatic biopsies on diagnosis stained with hematoxylin and eosin (H&E): right-sided (*A*—Gleason Score 8 (4 + 4)) and left-sided (*B*—Gleason Score 7 (4 + 3)) infiltration by acinar type prostatic adenocarcinoma. *B* shows areas of large cribriform Gleason pattern 4 adenocarcinoma (the absence of basal cells was confirmed with immunohistochemistry). (*C*,*D*) Rib biopsy during disease progression stained with H&E (*C*) and immunohistochemical staining (*D*). *C* shows areas of necrosis with hyperchromatic nuclei and little cytoplasm. In *D*, although staining for neuroendocrine markers CD56 (NCAM), synaptophysin, and chromogranin A was negative, TTF1 showed very focal nuclear positivity (arrow). Pan cytokeratin staining showed diffuse membrane positivity and prostate-specific antigen (PSA) (polyclonal) staining was negative. The features were of metastatic carcinoma, and, in view of the morphology, favored small-cell carcinoma.

A 63-yr-old previously well man presented with a history of weight loss and hip pain. The family history was relevant for breast cancer only with the patient's mother, maternal aunts, and sister having developed breast cancer. Blood tests demonstrated an elevated prostate-specific antigen (PSA) of 2900 µg/l (<4.0 µg/l), alkaline phosphatase (ALP) 2361 IU/l (44–147 IU/l) and a reduced Hb 10.8 g/dl (13.5–17.5g/dl). A computed tomography (CT) scan of his body and whole-body magnetic resonance imaging (MRI) imaging demonstrated a primary prostate cancer with bone and pulmonary metastases (Fig. 1A). A prostate biopsy confirmed adenocarcinoma with a Gleason score of 8 (4 + 4) (Fig. 2A,B). He was referred to oncology and started androgen-deprivation therapy (ADT) with bicalutamide and goserelin (Zoladex) injections.

**Figure 3.**
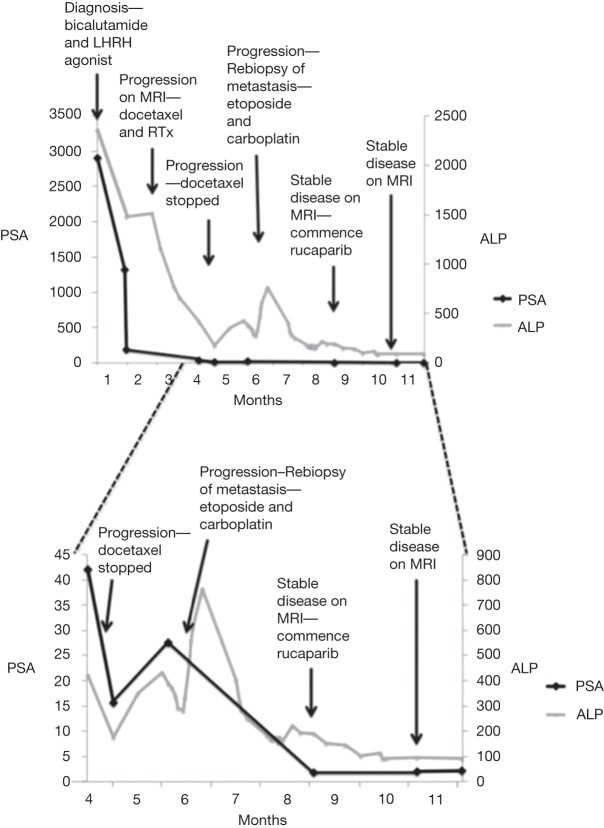
Timeline of prostate-specific antigen (PSA) and alkaline phosphatase (ALP) levels and management interventions from diagnosis. LHRH, leutinizing hormone-releasing hormone; MRI, magnetic resonance imaging.

The patient had an initial PSA response to testosterone deprivation, and within 3 mo the patient had gained weight with an unchanged performance status (PS) of 1. His PSA fell to 189.6 µg/l and alkaline phosphatase (ALP) to 1516 IU (Fig. 3). However, over the following weeks he clinically deteriorated with increasing pain in his right hip and upper leg. MRI imaging of his spine indicated progression of the bony metastases causing L4 nerve root compression (Fig. 1B). He was treated with five fractions of radiotherapy to his thoracic (20 Gy, five fractions) and lumbar (18 Gy, five fractions) spine and, given the radiographic progression, he commenced palliative docetaxel chemotherapy.

In the subsequent weeks, the patient's clinical condition deteriorated despite the docetaxel treatment. His PS quickly declined from 1 to 3. He experienced increasing pain from bony metastases and developed urinary retention requiring the insertion of a urinary catheter. Despite a fall in his ALP and PSA (Fig. 3), an MRI performed at this time indicated further progressive bony metastatic disease (Fig. 1C).

Given the rapid clinical progression was atypical for adenocarcinoma, a further biopsy was taken from an expanding rib metastasis. This demonstrated metastatic small-cell carcinoma positive for pan-cytokeratin (panCK) and thyroid transcription factor 1 (TTF1), but negative for other traditional markers of small-cell carcinoma such as 34BE12, CD56, synaptophysin, and chromogranin (Fig. 2C,D).

### Genomic Analysis

An established research programme at the Oxford Molecular Diagnostics Centre, a CPA (Clinical Pathology Accreditation; http://www.oxford-translational-molecular-diagnostics.org.uk) institute, aims to integrate genomic analysis into routine clinic care. WGS was discussed with the patient who consented to have both germline and tumor DNA sequencing performed from a fresh tumor biopsy.

**Figure 4.**
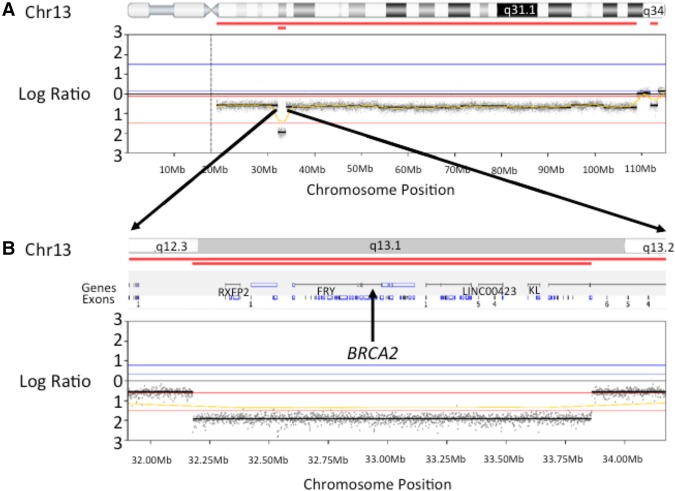
Log *R* plots generated using whole-genome sequencing read count information from tumor versus germline data: (*A*) Large regions of acquired copy-number (CN) loss involving Chr13 are identified in the tumor sample together with a ∼1.68-Mb region of acquired homozygous loss encompassing the *BRCA2* gene (highlighted, respectively, by the single and double red bars below the ideogram); (*B*) enlarged image of the acquired homozygous loss encompassing *BRCA2*.

Within the tumor, WGS identified two large genomic regions of copy neutral loss of heterozygosity, 41 genomic regions with acquired copy-number gains (10/41 with more than one additional copy) and 56 with acquired copy-number losses, including four separate regions displaying homozygous loss in copy number. One such homozygous loss was observed spanning ∼1.68 Mb of Chromosome 13 from 13q12.3-q13.1. This large homozygous deletion includes 21 separate genes or noncoding RNAs including *BRCA2* (Fig. 4). Despite the family history of breast cancer, no known pathogenic *BRCA1* or *BRCA2* germline variants were identified. A summary of the mutation analysis is shown in Table 1, and a more detailed variant table for the gene of interest, *BRCA2*, in Table 2.

**Table 1.** Mutation analysis of tumor genetic sequence

Gene	Mutation	Chromosome	Start	Finish
*CDC73*	CN gain	1	170,479,674	198,492,877
*VHL*	CN gain	3	0	16,153,088
*PDGFRA*	CN gain	4	52,652,733	65,798,728
*C-KIT*	CN gain	4	52,652,733	65,798,728
*NFKB*	CN loss	4	65,798,728	191,154,276
*FBXW7*	CN loss	4	65,798,728	191,154,276
*PIK3R*^a^	CN loss	5	49,405,693	97,198,579
*APC*	CN loss	5	101,904,635	130,674,136
*JAK2*	CN loss	9	64,626	10,139,240
*CDKN2A*	CN gain	9	10,320,113	26,205,565
*PTCH1*	CN gain	9	73,037,883	112,950,586
*NOTCH1*	CN gain	9	134,275,097	141,213,431
*PTEN*^a^	CN loss	10	85,557,432	105,804,295
*HRAS*	CN loss	11	0	11,638,440
*WT1*	CN gain	11	29,272,732	34,694,617
*KRAS*	CN loss	12	8,485,813	32,425,114
*FLT3*	CN loss	13	19,020,013	32,178,877
*BRCA2*	Homozygous copy loss	13	32,178,877	33,860,144
*RB1*	CN loss	13	33,860,144	109,025,409
*CDH1*	CN loss	16	46,455,960	90,354,753
*TP53*^a^	CN loss	17	7,506,837	7,671,804
*PAK7*	CN loss	20	0	14,782,559

Additional mutations that are commonly mutated in prostate cancer are identified through whole-genome sequencing (The Cancer Genome Atlas Research Network 2015).

CN, copy number.

^a^These are recognized mutations in primary prostate cancer.

**Table 2.** Summary of *BRCA2* mutation

Gene	Chromosome	HGVS DNA Reference	HGVS protein reference	Variant type	Predicted effect	dbSNP/dbVar ID	Genotype
*BRCA2*	13	g. 32178877–33860144del	n/a	Copy loss	Loss of function	n/a	Homozygous

HGVS, Human Genome Variation Society; dbSNP, Database for Short Genetic Variations; dbVar, Database of Genomic Structural Variation; n/a, not applicable.

### Treatment Outcome

Given the transition in histopathology from adenocarcinoma to small-cell carcinoma of the prostate the patient commenced a trial of etoposide and carboplatin chemotherapy. This resulted in a rapid improvement clinically, biochemically, (Fig. 3) and radiologically (Fig. 1D). Aside from treatment for neutropenic sepsis following the third cycle, the patient was able to complete six cycles of chemotherapy.

Tumors with mutations in genes involving homologous recombination such as *BRCA2* are sensitive to PARP inhibition, with a recent phase II study demonstrating biochemical, radiological, and survival benefits in patients with *BRCA2*-mutant prostate cancer (McCabe et al. 2006; Mateo et al. 2015). After completion of chemotherapy, the patient was started on the PARP inhibitor rucaparib, which was generously provided by Clovis Oncology for use on a compassionate basis outside of a clinical trial. The patient continues on rucaparib with clinical benefit at the time of submission (13 mo).

## DISCUSSION

This case highlights the potential value of rebiopsy and utility of WGS in personalized treatment of prostate cancer. The further biopsy of the rib lesion was performed in the face of progressive disease despite falling PSA levels, revealing a *BRCA2* deletion not present in the germline. To our knowledge, this is the first case study demonstrating the identification of a *BRCA2* deletion in dedifferentiated prostate cancer.

The dedifferentiation of cancers from adenocarcinoma to small-cell carcinoma is more commonly observed in *EGFR* mutated lung cancer and may confer sensitivity to platinum-based therapies (Sequist et al. 2011). Such dedifferentiation with small-cell recurrence has similarly been described in breast adenocarcinoma in a germline *BRCA2* mutation carrier (Niravath et al. 2015). The induction of neuroendocrine transformation in adenocarcinoma of the prostate is recognized and is associated with loss of tumor-suppressor genes (such as *TP53*, which was mutated in this case), loss of androgen-related pathways, and activation of mitotic pathways such as aurora kinase A thought to play significant roles (Li et al. 2013; Vlachostergios and Papandreou 2015). Typically this transformation is thought to occur after a more prolonged duration of androgen deprivation than reported here (Beltran et al. 2012). New guidelines regarding the immunohistochemical classification of small-cell carcinoma of the prostate emphasize the need for accurate classification of this aggressive subtype of prostate cancer, which is known to be particularly platinum-sensitive (Epstein et al. 2014).

WGS of the rib lesion found a homozygous deletion of *BRCA*2 leading to successful treatment with a PARP inhibitor. It is important to note that, despite the family history, this patient was not found to carry a known pathogenic *BRCA1* or *BRCA2* mutation, and this observation was only made through sequencing tumoral DNA. WGS promises to offer a new level of personalized therapy, as illustrated here and in other cases such as the recent use of WGS to identify BCL2 inhibitors as a therapeutic option for a patient with chronic myeloid leukemia (CML) resistant to ponatinib therapy (Korfi et al. 2015). Here we demonstrate that adoption of a lower threshold for repeat biopsy and consideration of WGS can have a significant and beneficial impact on patient treatment and outcomes. Although we would not advocate repeat biopsies and sequencing for all patients, our report suggests this may be particularly pertinent in cases with rapid progression on standard treatment or an unusual clinical phenotype. This study, among others, supports the notion that as the costs of WGS falls, clinicians should increasingly consider this approach as it may reveal avenues to personalized cancer treatment.

## METHODS

### DNA Extraction

Germline DNA was isolated from 1.5 ml peripheral blood using the QIASymphony DSP DNA Midi kit (QIAGEN), according to the manufacturer's protocol. Tumor DNA was extracted from fresh frozen tissue using the All Prep Mini DNA Extraction kit (QIAGEN), as described in the manufacturer's protocol.

### Library Preparation and Whole-Genome Sequencing

Libraries of 350-bp fragments were generated from 1 µg sheared genomic DNA using the TruSeq PCR-Free library preparation kit (Illumina). Of note, 2 × 126 bp paired-end sequencing was performed using the HiSeq2500 HTv4 (Illumina). WGS was performed at a coverage of 30× for the germline and of 75× for the tumor (Supplemental Table S1).

### Bioinformatic Data Analysis—SNV Calling

Paired-end alignment of sequencing data against the reference genome hg19 (GRCh 37) was performed using the Whole Genome Sequencing Application v2.0, based on the Isaac Alignment Tool (Raczy et al. 2013) within BaseSpace, a cloud-based analysis tool suit (Illumina).

Somatic single-nucleotide variant (SNV) and insertion/deletion (indel) variant calling analysis was performed using the Tumour-Normal Application v1.0, based on Strelka (Saunders et al. 2012), within BaseSpace. Calls were annotated using the Variant Effect Predictor v2.8 (McLaren et al. 2016), COSMIC v77 (Forbes et al. 2015), and 1000 Genomes v3 (The 1000 Genomes Project Consortium 2015). The SIFT (Kumar et al. 2009) and PolyPhen-2 (Adzhubei et al. 2010) algorithms were used to evaluate the impact of a mutation on protein structure or function as predicted by Ensembl (v84) (McLaren et al. 2016). All variants of interest were manually inspected using Integrative Genomics Viewer (IGV) (Robinson et al. 2011).

### Copy-Number Analysis—Structural Variants and Translocation Detection

Structural variant (SV) calling analysis was performed locally using BreakDancer [v1.4.5] (Chen et al. 2009). Analysis was limited to a set of disease-specific genes, as defined in Atlas of Genetics and Cytogenetics in Oncology and Haematology (Huret et al. 2013), accessed at the time of analysis.

The coverage of the aligned sequence data was determined by the total number of aligned bases divided by the genome size. This information is provided by the Whole Genome Sequencing Application v2.0 (Raczy et al. 2013).

Manual inspection in IGV is essential for each of the translocations reported by BreakDancer in order to view the complexity and quality of the sequence alignment of the regions that were reported and to verify whether the event is somatic or germline.

### Clinical Interpretation

All SNVs, indels, and copy-number variants (CNVs) identified within the COSMIC Cancer Genes Census (v77) (Forbes et al. 2015) ranked with respect to their pathogenicity and clinical actionability (Futreal et al. 2004). A number of different sources, including COSMIC (Forbes et al. 2015), “MyCancerGenome” (www.mycancergenome.org), and ClinicalTrials.gov (www.clinicaltrials.gov) were used to determine whether genetic alterations were clinically relevant.

Table 1 and Supplemental Table S2 contain details of this analysis.

## ADDITIONAL INFORMATION

### Data Deposition and Access

The reported variants are deposited in the ClinVar database (http://www.ncbi.nlm.nih.gov/clinvar/) under accession numbers SCV000493829, SCV000493830, and SCV000493831.

### Ethics Statement

Written consent for this case study, sequencing, and publication thereof was obtained from the patient and ethical approval granted in line with the Biomedical Research Centre (BRC) Cancer Pilot (Ref: 14/SC/1165 South Central Berkshire B REC). Written consent did not include WGS archiving or raw data deposition.

### Acknowledgments

We thank the patient for his involvement and agreement to us sharing this case.

### Author Contributions

K.P. and B.P.F. wrote and revised the manuscript, and K.P. created the table and figures. A.S., S.K., P.A., H.D., N.P., and J.C.T. performed the WGS and subsequent analysis. A.P. edited the manuscript and was involved in the clinical management of the patient. I.R., L.B., Z.T., D.K., C.V., K.G., and M.T. were involved in the clinical diagnosis and management of the patient.

### Funding

This study was funded by the Wellcome Trust and Department of Health as part of the Health Innovation Challenge Fund. The views expressed in this manuscript are those of the authors and not necessarily those of the Wellcome Trust and Department of Health. B.P.F. was a National Institute for Health Research (NIHR)-funded Clinical Lecturer and is a Wellcome Trust Funded Intermediate Clinical Fellow (ref:201488/Z/16/Z). K.P. is an NIHR-funded Academic Clinical Fellow.

### Competing Interest Statement

The authors have declared no competing interest.

## Supplementary Material

Supplemental Material
