# Supplementary material for: Whole-genome sequencing identifies homozygous BRCA2 deletion guiding treatment in dedifferentiated prostate cancer
Source: Cold Spring Harb Mol Case Stud. 2017 May;3(3):a001362. doi: 10.1101/mcs.a001362 (PMC5411692; doi:10.1101/mcs.a001362)
Supplement: Supplemental Material [file supp_3_3_a001362__index.html]

Whole-genome sequencing identifies homozygous BRCA2 deletion guiding treatment in dedifferentiated prostate cancer — Supplemental Material 

# Whole-genome sequencing identifies homozygous *BRCA2* deletion guiding treatment in dedifferentiated prostate cancer

## Supplemental Material

- Supplemental\_Table\_S1.docx
- Supplemental\_Table\_S2.pdf
