# Supplementary material for: Whole-genome sequencing identifies homozygous BRCA2 deletion guiding treatment in dedifferentiated prostate cancer
Source: Cold Spring Harb Mol Case Stud. 2017 May;3(3):a001362. doi: 10.1101/mcs.a001362 (PMC5411692; doi:10.1101/mcs.a001362)
Supplement: Supplemental Material [file supp_mcs.a001362_Supplemental_Table_S1.docx]

**Supplementary Table S1 -** Whole Genome Sequencing (WGS) coverage and mapped reads

• Germline – WGS Average Coverage: 27.40x

General Information

| **Total Reads** | **Mapped Reads** | **% Mapped reads** | **Properly Paired Reads** | **% Properly Paired Reads** |
| --- | --- | --- | --- | --- |
| 722,500,052 | 690,234,773 | 95.3 | 665,067,106 | 92.0 |

Coverage Information over the Whole Exome

| **Average Coverage** | **% Bases above 10x** | **% Bases above 20x** | **% Bases above 30x** |
| --- | --- | --- | --- |
| 28.12x | 98.2 | 86.5 | 42.7 |

• Tumour – WGS Average Coverage: 85.44x

General Information

| **Total Reads** | **Mapped Reads** | **% Mapped reads** | **Properly Paired Reads** | **% Properly Paired Reads** |
| --- | --- | --- | --- | --- |
| 2,278,844,976 | 2,185,930,417 | 95.92 | 2,084,723,948 | 91.48 |

Coverage Information over the Whole Exome

| **Average Coverage** | **% Bases above 10x** | **% Bases above 20x** | **% Bases above 30x** |
| --- | --- | --- | --- |
| 89.5 | 99.2 | 98.8 | 98.2 |
