# Supplementary material for: Whole-genome sequencing identifies homozygous BRCA2 deletion guiding treatment in dedifferentiated prostate cancer
Source: Cold Spring Harb Mol Case Stud. 2017 May;3(3):a001362. doi: 10.1101/mcs.a001362 (PMC5411692; doi:10.1101/mcs.a001362)
Supplement: Supplemental Material [file supp_mcs.a001362_Supplemental_Table_S2.pdf]

Supplementary Table S2 - Summary of mutations identified by WGS

Chr	Start	End	Acquired Event	Length (bp)	Cytoband	Gene Symbols	OncoScan SomMut Markers
1	0	26,138,050	CN Loss	26138051	p36.33 - p36.11	DDX11L1, MIR6859-1, MIR6859-2, WASH7P, FAM138A, FAM138F, OR4F5, LOC729737, DQ597235, DQ599768, LOC100133331, LOC100132062, LOC100132287, LOC388312, OR4F16, OR4F29, BC036251, JA429830, JA429831, JB137814, MIR6723, M37726, OR4F29, OR4F3, DQ575786, DQ599872, LOC100133331, AK310751, LOC100288069, FAM87B, LINC00115, LINC01128, LOC643837, FAM41C, AK056486, LOC100130417, SAMD11, NOC2L, KLHL17, PLEKHN1, C1orf170, PERM1, HES4, ISG15, AGRN, AK310350, BC033949, RNF223, C1orf159, LOC254099, MIR200B, MIR200A, JA715134, MIR429, JA715143, AK128833, TTLL10, TNFRSF18, TNFRSF4, SDF4, SDF4, B3GALT6, FAM132A, UBE2J2, SCNN1D, MIR6726, ACAP3, PUSL1, MIR6727, CPSF3L, GLTPD1, TAS1R3, MIR6808, DVL1, MXRA8, AURKAIP1, CCNL2, LOC148413, MRPL20, ANKRD65, TMEM88B, VWA1, ATAD3C, ATAD3B, ATAD3A,	0

1	27,148,195	28,578,367	CN Loss	1430173	p36.11 - p35.3	ZDHHC18, SFN, GPN2, GPATCH3, NR0B2, NUDC, BC016143, KDF1, C1orf172, TRNP1, FAM46B, SLC9A1, DL490887, WDTC1, LOC644961, TMEM222, SYTL1, MAP3K6, FCN3, CD164L2, GPR3, WASF2, AHDC1, FGR, IFI6, FAM76A, STX12, SCARNA1, PPP1R8, THEMIS2, RPA2, SMPDL3B, XKR8, EYA3, PTAFR, DNAJC8, ATPIF1, JA611241		0

1	170,479,674	198,492,877	CN Gain	28013204	q24.2 - q31.3	AK096329, GORAB, PRRX1, MROH9, MIR1295A, MIR1295B, FMO3, FMO6P, FMO2, FMO1, FMO4, TOP1P1, PRRC2C, MYOC, VAMP4, METTL13, AK094818, DNM3, MIR3120, MIR214, DNM3OS, MIR199A2, PIGC, C1orf105, SUCO, FASLG, TNFSF18, TNFSF4, LOC100506023, BC136808, PRDX6, SLC9C2, LOC730159, ANKRD45, KLHL20, CENPL, DARS2, GAS5-AS1, SNORD81, SNORD47, SNORD80, SNORD79, SNORD78, GAS5, SNORD44, SNORD77, SNORD76, SNORD75, SNORD74, ZBTB37, DQ593451, SERPINC1, RC3H1, GPR52, RABGAP1L, CACYBP, MRPS14, TNN, KIAA0040, TNR, AK093214, BC043291, SCARNA3, RFWD2, PAPPA2, AK096718, ASTN1, MIR488, FAM5B, BRINP2, LOC101928778, SEC16B, LOC730102, RASAL2-AS1, RASAL2, TEX35, C1orf220, MIR4424, RALGPS2, ANGPTL1, FAM20B, TOR3A, ABL2, DD413682, SOAT1, AXDND1, NPHS2, DQ592690, DQ571986, DQ600552, DQ595741, DQ601755,	CDC73_pW43X_c128G_A

1	198,492,878	230,986,905	High Copy Gain	32494028	q31.3 - q42.2	ATP6V1G3, PTPRC, MIR181B1, MIR181A1, MIR181A1HG, LINC01222, BC040869, NR5A2, LINC00862, ZNF281, EU250746, KIF14, DDX59, LOC101929224, CAMSAP2, GPR25, C1orf106, C1orf81, KIF21B, BC016656, CACNA1S, ASCL5, TMEM9, IGFN1, PKP1, AK055533, TNNT2, TNNT2, LAD1, TNNI1, PHLDA3, CSRP1, RPS10P7, AX747377, MIR5191, NAV1, IPO9-AS1, RNU6-79P, MIR1231, IPO9, MIR6739, SHISA4, LMOD1, TIMM17A, RNPEP, MIR6740, ELF3, GPR37L1, ARL8A, PTPN7, PTPRVP, LGR6, UBE2T, PPP1R12B, SYT2, KDM5B, PCAT6, KDM5B-AS1, LOC641515, BC040684, BC049825, LOC148709, RABIF, KLHL12, ADIPOR1, CYB5R1, LOC401980, LOC100506747, TMEM183B, TMEM183A, PPFIA4, MYOG, ADORA1, MYBPH, BC034684, CHI3L1, CHIT1, LOC100506775, LINC01136, LOC730227, BTG2, FMOD, PRELP, OPTC, ATP2B4, U42379, SNORA77, LINC00260, LAX1, ZBED6, ZC3H11A, SNRPE, LINC00303, SOX13, ETNK2, BC038769, REN, KISS1,	0

1	230,986,906	241,946,332	CN Gain	10959427	q42.2 - q43	C1orf198, LOC101927604, BC032911, TTC13, ARV1, MIR1182, FAM89A, LOC149373, TRIM67, C1orf131, GNPAT, EXOC8, SPRTN, EGLN1, SNRPD2P2, TSNAX, LINC00582, TSNAX-DISC1, DISC2, DISC1, SIPA1L2, LOC101927683, MAP10, NTPCR, PCNXL2, KIAA1804, MIR4427, KCNK1, SLC35F3, AK054726, MIR4671, COA6, TARBP1, LOC100506795, IRF2BP2, LINC00184, BC032040, LINC01132, LOC100506810, BC016972, TOMM20, SNORA14B, RBM34, MIR4753, ARID4B, GGPS1, TBCE, B3GALNT2, AX747026, GNG4, LYST, MIR1537, NID1, AX747246, GPR137B, ERO1LB, EDARADD, LGALS8-AS1, LGALS8, HEATR1, ACTN2, MTR, MT1HL1, RYR2, ZP4, LOC100130331, LINC01139, LOC339535, CHRM3, CHRM3-AS2, CHRM3-AS1, RPS7P5, FMN2, GREM2, RGS7, MIR3123, FH, KMO, OPN3, CHML, WDR64		0
1	241,946,333	242,628,315	cnLOH	681983	q43	WDR64, EXO1, BECN1P1, MAP1LC3C, PLD5		0
1	242,628,316	244,221,452	CN Gain	1593137	q43 - q44	PLD5, LOC731275, Mir_350, CEP170, MIR4677, SDCCAG8, AKT3, LOC339529, ZBTB18, AK310634		0

1	244,221,453	244,423,458	High Copy Gain	202006	q44	0	0
2	14,057,047	14,076,539	CN Loss	19493	p24.3	0	0
2	103,694,579	103,752,839	CN Loss	58261	q12.1	0	0
2	103,909,636	104,084,950	CN Loss	175315	q12.1	0	0
2	104,172,062	168,223,828	CN Loss	64051767	q12.1 - q24.3	LOC100287010, LINC01102, LOC150568, LINC01103, LINC01114, LOC284998, LINC01158, LOC100506421, POU3F3, AK095498, LINC01159, LOC102724691, MRPS9, LOC101927492, GPR45, LOC644617, TGFBRAP1, C2orf49, FHL2, LOC285000, NCK2, C2orf40, UXS1, PLGLA, RGPD3, ST6GAL2, RGPD4-AS1, LOC729121, RGPD4, SLC5A7, SULT1C3, SULT1C2, SULT1C2P1, SULT1C4, GCC2, FLJ38668, LIMS1, RANBP2, CCDC138, EDAR, SH3RF3-AS1, MIR4265, MIR4266, SH3RF3, SEPT10, AX747172, SOWAHC, RGPD5, RGPD6, LIMS3L, LIMS3, LIMS3-LOC440895, LOC440895, LOC100288570, DQ584199, LINC01123, LOC440894, MIR4267, MIR4436B1, MIR4436B2, MALL, NPHP1, LINC00116, LOC100507334, DQ595602, DQ587734, MIR4436B1, LINC01106, LOC151009, LINC01123, DQ586666, DQ581602, LOC440895, LIMS3-LOC440895, LIMS3L, RGPD5, BUB1, DQ594093, DQ573754, DQ593945, DQ574014, DQ592678,	0
2	235,595,691	235,675,218	CN Gain	79528	q37.1 - q37.2	0	0

3	0	16,153,088	CN Gain	16153089	p26.3 - p25.1	LOC102723448, CHL1, CHL1-AS1, BC065754, AK126307, LINC01266, CNTN6, CNTN4-AS2, CNTN4, IL5RA, TRNT1, CRBN, BC141932, LRRN1, SUMF1, SETMAR, ITPR1-AS1, ITPR1, EGOT, BHLHE40-AS1, BHLHE40, ARL8B, EDEM1, MIR4790, Metazoa_SRP, AF279782, LOC101927347, GRM7, LOC101927394, AK124857, LMCD1-AS1, U4atac, LMCD1, DQ591848, LINC00312, AX747864, SSUH2, CAV3, OXTR, Mir_548, RAD18, SRGAP3, BC041457, SRGAP3-AS3, SETD5-AS1, THUMPD3, THUMPD3-AS1, SETD5, LHFPL4, MTMR14, CPNE9, BRPF1, OGG1, AX748417, CAMK1, TADA3, ARPC4, TTLL3, ARPC4-TTLL3, RPUSD3, JAGN1, IL17RE, IL17RC, CRELD1, CIDEC, PRRT3, PRRT3-AS1, EMC3, AX747493, AK125558, EMC3-AS1, LOC401052, FW339974, CIDECP, FANCD2, FANCD2OS, BRK1, VHL, IRAK2, TATDN2, LINC00852, GHRLOS, GHRL, SEC13, MIR885, ATP2B2, LINC00606, SLC6A11, SLC6A1-AS1, SLC6A1, HRH1, ATG7, AX748267,	VHL_p_c463_plus_2T_C, VHL_p_c464_minus_1G_A, VHL_pP61P_c183C_T, VHL_pS65L_c194C_T, VHL_pS68X_c203C_A, VHL_pP81S_c241C_T, VHL_pL85P_c254T_C, VHL_pW88X_c263G_A, VHL_pQ96X_c286C_T, VHL_pG114C_c340G_T, VHL_pH115Y_c343C_T, VHL_pW117R_c349T_C, VHL_pW117X_c350G_A, VHL_pL118P_c353T_C, VHL_pQ132X_c394C_T, VHL_pI151S_c452T_G, VHL_pL153P_c458T_C, VHL_p_c463_plus_1G_T, VHL_pE160K_c478G_A, VHL_pR161X_c481C_T, VHL_pR167W_c499C_T, VHL_pS183X_c548C_A, VHL_pL184P_c551T_C, VHL_pE189K_c565G_A
3	125,714,676	125,818,900	CN Gain	104225	q21.2 - q21.3	SLC41A3	0

3	125,818,900	127,636,899	High Copy Gain	1818000	q21.3	SLC41A3, ALDH1L1-AS1, ALDH1L1, ALDH1L1-AS2, KLF15, BC033989, CCDC37-AS1, CCDC37, UNQ2790, ZXDC, UROC1, CHST13, C3orf22, TXNRD3NB, TXNRD3, NUP210P1, CHCHD6, PLXNA1, C3orf56, LOC101927123, BC015846, BX537548, MIR6825, TPRA1, MCM2, PODXL2, ABTB1, MGLL, KBTBD12		0
3	127,636,900	128,042,606	CN Loss	405707	q21.3	KBTBD12, SEC61A1, RUVBL1, EEFSEC		0

4	52,652,733	65,798,728	CN Gain - includes *REST*	13145996	q11 - q13.1	DCUN1D4, LRRC66, SGCB, SPATA18, USP46, MIR4449, DANCR, SNORA26, ERVMER34-1, LOC152578, RASL11B, BC042091, AK055055, SCFD2, FIP1L1, LNX1-AS1, LNX1, LNX1-AS2, PDGFRA, RPL21P44, CHIC2, GSX2, BC044946, KIT, DL490879, KDR, Metazoa_SRP, SRD5A3, SRD5A3-AS1, TMEM165, CLOCK, PDCL2, NMU, U6, LOC644145, EXOC1, CEP135, KIAA1211, AASDH, PPAT, PAICS, SRP72, ARL9, THEGL, Mir_720, HOPX, SPINK2, REST, NOA1, POLR2B, IGFBP7, LOC255130, IGFBP7-AS1, BC034799, LPHN3, Y_RNA, BC039452, LOC101927186, TECRL, LOC401134	PDGFRA_pN659K_c1977C_A, PDGFRA_pT674I_c2021C_T, PDGFRA_pF808L_c2422T_C, PDGFRA_pV824V_c2472C_T, PDGFRA_pD842Y_c2524G_T, PDGFRA_pD842V_c2525A_T_allele1, PDGFRA_pD846Y_c2536G_T, PDGFRA_pN870S_c2609A_G, PDGFRA_pD1071N_c3211G_A, KIT_pV560D_c1679T_A_allele1, KIT_pL576P_c1727T_C, KIT_pF584S_c1751T_C, KIT_pD52N_c154G_A, KIT_pY503_F504insAY_c1509_1510insGCCTAT_allele1, KIT_pW557_K558del_c1669_1674delTGGAAG_allele1, KIT_pW557R_c1669T_C, KIT_pV559A_c1676T_C, KIT_pP585P_c1755C_T, KIT_pK642E_c1924A_G, KIT_pV654A_c1961T_C, KIT_pT670I_c2009C_T, KIT_pI798I_c2394C_T, KIT_pD816Y_c2446G_T, KIT_pN822K_c2466T_G, KIT_pY823D_c2467T_G, KIT_pV825A_c2474T_C,

4	65,798,728	191,154,276	CN Loss	125355549	q13.1 - q35.2	LOC401134, EPHA5, LOC100144602, TRNA, LOC101927237, AK093203, CENPC, CENPC1, STAP1, UBA6, UBA6-AS1, BC045560, GNRHR, TMPRSS11D, LOC550112, TMPRSS11A, TMPRSS11GP, SYT14L, LOC550113, TMPRSS11F, FTLP10, TMPRSS11BNL, TMPRSS11B, YTHDC1, TMPRSS11E, UGT2B17, UGT2B15, UGT2B10, UGT2A3, UGT2B7, UGT2B11, AK124272, UGT2B28, UGT2B4, UGT2A2, UGT2A1, SULT1B1, SULT1E1, CSN1S1, CSN2, STATH, HTN3, HTN1, CSN1S2AP, CSN1S2BP, C4orf40, PRR27, ODAM, FDCSP, CSN3, CABS1, SMR3A, SMR3B, PROL1, MUC7, AMTN, AMBN, ENAM, IGJ, UTP3, RUFY3, GRSF1, MOB1B, DCK, SLC4A4, GC, NPFFR2, ADAMTS3, COX18, ANKRD17, ALB, AFP, AFM, LOC728040, RASSF6, IL8, CXCL8, CXCL6, PF4V1, CXCL1, PF4, PPBP, CXCL5, CXCL3, PPBPP2, CXCL2, LOC541467, MTHFD2L, BC016361, EPGN, EREG, AREG, BTC, AK027257, PARM1, LOC441025, RCHY1, THAP6, C4orf26, CDKL2, G3BP2, AK311578,	NFKB1_p_c40_minus_1G_A, FBXW7_pS582L_c1745C_T, FBXW7_pR505C_c1513C_T, FBXW7_pR479Q_c1436G_A, FBXW7_pR465H_c1394G_A, FBXW7_pR465C_c1393C_T, FBXW7_pR393X_c1177C_T, FBXW7_pR278X_c832C_T, FBXW7_pR224X_c670C_T

5	1	46,427,438	cnLOH	46427438	p15.33 - p11	PLEKHG4B, LRRC14B, CCDC127, SDHA, LOC102467073, PDCD6, AHRR, C5orf55, EXOC3, FLJ00157, AK023178, PP7080, BC013821, SLC9A3, MIR4456, LOC100996325, CEP72, TPPP, ZDHHC11, BRD9, TRIP13, LOC100506688, NKD2, MIR4635, SLC12A7, CTD-3080P12.3, BC032469, SLC6A19, SLC6A18, TERT, MIR4457, AK126225, CLPTM1L, DQ598099, BC034612, SLC6A3, LPCAT1, MIR6075, CR749689, SDHAP3, LOC728613, MIR4277, MRPL36, NDUFS6, LOC101929034, IRX4, CTD-2194D22.4, MIR548BA, LOC100506858, IRX2, C5orf38, LOC102467074, LINC01019, LOC285577, LOC102467075, IRX1, LOC101929153, BC034630, LINC01020, LOC340094, AK094462, CTD-2297D10.2, ADAMTS16, KIAA0947, FLJ33360, MED10, UBE2QL1, LINC01018, LOC255167, NSUN2, SRD5A1, LOC100505625, PAPD7, MIR4278, MIR4454, LOC442132, ADCY2, C5orf49, FASTKD3, MTRR, LOC729506,	0

5	49,405,693	97,198,579	CN Loss	47792887	q11.1 - q15	EMB, PARP8, LOC100287592, LOC642366, ISL1, PELO, ITGA1, AK097288, ITGA2, MOCS2, LOC257396, FST, NDUFS4, BC048141, MIR581, Metazoa_SRP, ARL15, HSPB3, SNX18, LOC102467080, ESM1, LOC102467081, GZMK, Y_RNA, GZMA, CDC20B, GPX8, MIR449A, MIR449B, MIR449C, MCIDAS, CCNO, DHX29, SKIV2L2, PPAP2A, MIR5687, RNF138P1, SLC38A9, DDX4, IL31RA, IL6ST, FLJ31104, U6, ANKRD55, U6atac, LOC102467147, LOC101928448, MAP3K1, SETD9, MIER3, GPBP1, ACTBL2, LOC101928505, LOC101928539, LOC101928569, Mir_562, PLK2, GAPT, LOC101928600, RAB3C, AB074188, BX641110, Mir_582, PDE4D, PART1, DEPDC1B, ELOVL7, ERCC8, NDUFAF2, SMIM15, CTC-436P18.1, ZSWIM6, BC032910, C5orf64, BC043229, BC043516, LOC101928651, BC043261, BC039381, LOC100506526, KIF2A, DIMT1, IPO11, LRRC70, IPO11-LRRC70, HTR1A, RNF180, RGS7BP, FAM159B,	PIK3R1_pG376R_c1126G_A
5	97,198,579	101,904,635	Homozygous Copy Loss	4706057	q15 - q21.1	HM358988, RGMB-AS1, RGMB, CHD1, LOC100289230, DQ597441, CTD-2151A2.1, JB137812, LOC100133050, DQ583509, FAM174A, ST8SIA4, SLCO4C1, SLCO6A1	0

5	101,904,635	130,674,136	CN Loss	28769502	q21.1 - q31.1	LINC00492, AX747345, LINC00491, PAM, GIN1, PPIP5K2, C5orf30, LOC102467212, NUDT12, RAB9BP1, LOC102467213, EFNA5, FBXL17, LINC01023, HP07349, BC034788, FER, AK021888, JB023059, PJA2, MAN2A1, LOC100289673, TMEM232, SNORA51, SLC25A46, TSLP, WDR36, CAMK4, STARD4, STARD4-AS1, NREP, NREP-AS1, EPB41L4A-AS1, SNORA13, LOC101927023, EPB41L4A, FLJ11235, EPB41L4A-AS2, LOC102467214, LOC102467216, APC, SRP19, ZRSR1, REEP5, DCP2, MCC, FLJ43978, TSSK1B, U4atac, YTHDC2, KCNN2, AK097686, TRIM36, PGGT1B, CCDC112, FEM1C, TICAM2, TMED7-TICAM2, LOC101927100, TMED7, LOC102467217, CDO1, ATG12, AP3S1, AX747550, AQPEP, ARL14EPL, COMMD10, LOC101927190, CTB-118N6.3, SEMA6A, Mir_633, LOC102467223, LINC00992, LOC728342, LOC102467224, BC044609, LOC100505811, LOC101927280, LOC102467225, DTWD2, MIR1244-1, MIR1244-2,	APC_pR213X_c637C_T, APC_pR232X_c694C_T, APC_p_c835_minus_8A_G, APC_pR283X_c847C_T, APC_pR302X_c904C_T, APC_pR332X_c994C_T, APC_pR564X_c1690C_T, APC_pQ789X_c2365C_T, APC_pE853X_c2557G_T, APC_pR876X_c2626C_T, APC_pR1114X_c3340C_T, APC_pS1281X_c3842C_A, APC_pE1286X_c3856G_T, APC_pQ1291X_c3871C_T, APC_pQ1294X_c3880C_T, APC_pE1309fs4_c3921_3925delAAAAG_allele1, APC_pE1309fs6_c3923_3924insA_allele1, APC_pE1309X_c3925G_T, APC_pE1309fs4_c3927_3931delAAAGA_allele1, APC_pE1322X_c3964G_T, APC_pQ1328X_c3982C_T, APC_pQ1338X_c4012C_T, APC_pS1341R_c4023T_G, APC_pE1345X_c4033G_T, APC_pE1353X_c4057G_T, APC_pQ1367X_c4099C_T,
6	57,202,434	58,140,638	CN Loss	938205	p11.2	PRIM2	0
6	61,880,946	63,017,763	CN Loss	1136818	q11.1	MTRNR2L9, KHDRBS2	0
6	70,162,067	70,188,348	CN Loss	26282	q13	0	0
6	72,089,455	72,104,503	CN Loss	15049	q13	0	0

6	72,890,949	74,093,387	CN Loss	1202439	q13	RIMS1, KCNQ5-IT1, KCNQ5, MIR4282, KCNQ5-AS1, KHDC1L, KHDC1, BC031876, AL832252, C6orf147, DPPA5, KHDC3L, OOEP	0
6	74,417,601	75,787,443	CN Loss	1369843	q13	CD109, LOC101928516, AF086303	0
6	75,896,327	75,965,932	CN Loss	69606	q13 - q14.1	COL12A1, COX7A2, TMEM30A	0
6	76,195,729	77,436,689	CN Loss	1240961	q14.1	FILIP1, SENP6, MYO6, IMPG1	0
6	77,456,848	78,940,754	CN Loss	1483907	q14.1	HTR1B, MEI4	0
6	90,314,978	90,348,595	CN Loss	33618	q15	ANKRD6, LYRM2, LOC101929057	0
6	96,666,569	103,740,578	CN Gain	7074010	q16.1 - q16.3	UFL1, FHL5, U4, GPR63, NDUFAF4, Metazoa_SRP, KLHL32, MMS22L, MIR548H3, AK091365, LOC101927314, MIR2113, POU3F2, FBXL4, FAXC, COQ3, BC033061, PNISR, DQ599242, USP45, TSTD3, CCNC, PRDM13, MCHR2, MCHR2-AS1, LOC728012, SIM1, ASCC3, DQ585302, GRIK2	0

6	103,762,789	118,438,903	CN Gain	14676115	q16.3 - q22.2	HACE1, LINC00577, LIN28B, BVES, BVES-AS1, POPDC3, PREP, PRDM1, ATG5, AIM1, RTN4IP1, QRSL1, LOC100422737, AK123416, C6orf203, BEND3, PDSS2, SOBP, SCML4, SEC63, AJ420489, OSTM1, AF520419, NR2E1, SNX3, LACE1, FOXO3, LINC00222, ARMC2, ARMC2-AS1, SESN1, CEP57L1, LOC100996634, CCDC162P, C6orf185, CD164, PPIL6, SMPD2, MICAL1, ZBTB24, AK9, AK9, FIG4, GPR6, WASF1, CDC40, METTL24, DDO, SLC22A16, CDK19, BC047513, AMD1, GTF3C6, RPF2, GSTM2P1, SLC16A10, KIAA1919, REV3L, AF216583, TRAF3IP2-AS1, TRAF3IP2, FYN, WISP3, TUBE1, FAM229B, LAMA4, RFPL4B, MARCKS, LINC01268, LOC285758, FLJ34503, HDAC2, BC042098, Mir_320, HS3ST5, Mir_548, Mir_584, FRK, TPI1P3, COL10A1, NT5DC1, TSPYL4, AK093256, TSPYL1, DSE, FAM26F, FAM26E, TRAPPC3L, FAM26D, RWDD1, RSPH4A, ZUFSP, AX746765, KPNA5, FAM162B, GPRC6A, RFX6, VGLL2, ROS1, GOPC, DCBLD1,	0
6	118,489,760	119,286,553	CN Gain	796794	q22.2 - q22.31	SLC35F1, BRD7P3, PLN, CEP85L, LOC100287632, MCM9, ASF1A, FAM184A	0
6	119,321,071	120,141,501	CN Gain	820431	q22.31	FAM184A, MIR548B, MAN1A1, LOC285762	0
6	120,157,874	120,358,108	CN Gain	200235	q22.31	0	0

6	120,369,437	120,929,676	CN Gain	560240	q22.31	0	0
7	27,961,464	37,358,056	CN Loss	9396593	p15.2 - p14.1	JAZF1, U6, JAZF1-AS1, BC087859, CREB5, TRIL, DQ601810, LOC100506497, CPVL, LOC101928168, LOC102724484, CHN2, Mir_340, BC038570, PRR15, BC121815, LOC646762, MIR550A3, ZNRF2P2, DPY19L2P3, WIPF3, SCRN1, FKBP14, PLEKHA8, MTURN, C7orf41, AK098769, MIR550A1, MIR550B1, ZNRF2, DKFZP586I1420, LINC01176, NOD1, GGCT, LOC401320, AL137445, DJ031144, GARS, CRHR2, INMT, INMT-FAM188B, FAM188B, AQP1, GHRHR, ADCYAP1R1, NEUROD6, CCDC129, PPP1R17, BC043188, Mir_584, PDE1C, LOC100130673, LSM5, DPY19L1P1, ZNRF2P1, MIR550A2, MIR550B2, AK057321, LINC00997, LOC401321, AVL9, DPY19L1P2, KBTBD2, RP9P, FKBP9, AX721280, NT5C3A, DQ584906, RP9, 5S_rRNA, BBS9, hCG_1643653, AK025321, BMPER, NPSR1-AS1, NPSR1, AJ011981, DPY19L1, BC084560, DPY19L2P1, TBX20, LOC401324, HERPUD2, DQ594967, LOC100506725, LOC101928545,	0
7	117,729,741	117,742,473	CN Loss	12733	q31.31	0	0

8	0	24,252,431	CN Loss	24252432	p23.3 - p21.2	OR4F21, RPL23AP53, ZNF596, FAM87A, FBXO25, AK056623, TDRP, C8orf42, AK128400, ERICH1, ERICH1-AS1, LOC286083, DLGAP2-AS1, DLGAP2, AK093412, CLN8, MIR596, BC047307, ARHGEF10, KBTBD11, MIR7160, MYOM2, AX747124, BC045738, AK128880, CSMD1, 7SK, LOC100287015, MCPH1, ANGPT2, MIR8055, AGPAT5, MIR4659A, MIR4659B, XKR5, LOC100652791, GS1-24F4.2, DEFB1, DEFA6, DEFA4, DEFA8P, DEFA9P, DEFA10P, DEFA1, DEFA1B, DEFT1P, DEFT1P2, DEFA3, DEFA1B, DEFA11P, DEFA5, BC030294, FLJ00326, LINC00965, DEFB109P1B, FAM66B, USP17L1P, USP17L4, ZNF705G, DEFB4B, DEFB103A, DEFB103B, SPAG11B, DEFB104B, DEFB106A, DEFB106B, DEFB105A, DEFB105B, DEFB107A, DEFB107B, PRR23D1, PRR23D2, FAM90A7P, FAM90A10P, DEFB104A, DEFB104B, SPAG11A, DEFB103A, DEFB4A, ZNF705B, USP17L8, USP17L3, FAM66E, MIR548I3, FAM86B3P, SGK223, CLDN23, MFHAS1, ERI1,	0
8	30,121,907	30,138,226	CN Loss	16320	p12	0	0

8	62,312,437	70,435,154	CN Gain	8122718	q12.3 - q13.2	CLVS1, ASPH, MIR4470, BC047540, NKAIN3, UG0898H09, GGH, TTPA, LOC101410533, YTHDF3-AS1, YTHDF3, AK093370, LINC01289, LOC286184, LOC100130155, MIR124-2, MIR124-2HG, BX537900, LOC401463, BHLHE22, CYP7B1, LINC00251, LINC01299, LOC286186, ARMC1, MTFR1, TRNA_Tyr, PDE7A, DNAJC5B, TRNA_Tyr, TRNA_Ala, TRIM55, CRH, LINC00967, LOC100505659, RRS1-AS1, LOC100505676, RRS1, ADHFE1, C8orf46, MYBL1, LOC645895, VCPIP1, C8orf44, C8orf44-SGK3, PTTG3P, SGK3, MCMDC2, SNORD87, SNHG6, TCF24, PPP1R42, U2, JA611241, COPS5, CSPP1, ARFGEF1, CPA6, DQ584699, BC036055, PREX2, AX747593, LOC286189, C8orf34, 5S_rRNA, LOC100505718, LOC100505739, SULF1	0
8	70,435,155	73,497,200	High Copy Gain	3062046	q13.2 - q13.3	SULF1, SLCO5A1, PRDM14, NCOA2, LOC101926892, TRAM1, LOC286190, LACTB2, Metazoa_SRP, XKR9, EYA1, Mir_548, BC048982, MSC, LOC100132891, RNU6-83P, TRPA1, LOC392232, KCNB2	0

8	73,497,201	97,721,203	CN Gain	24224003	q13.3 - q22.1	KCNB2, LOC101926908, TERF1, SBSPON, LOC100130301, RPL7, RDH10, AK128216, STAU2-AS1, STAU2, UBE2W, TCEB1, TMEM70, LY96, JPH1, GDAP1, MIR5681A, MIR5681B, FLJ39080, MIR2052, PI15, AK024242, CRISPLD1, BC062758, CASC9, HNF4G, LINC01111, ZFHX4-AS1, ZFHX4, PEX2, BC036404, PKIA, ZC2HC1A, LOC101241902, IL7, STMN2, HEY1, U7, LOC101927040, AK055332, MRPS28, TPD52, MIR5708, DJ031142, ZBTB10, Mir_562, ZNF704, PAG1, FABP5, PMP2, FABP9, FABP4, FABP12, IMPA1, SLC10A5, ZFAND1, CHMP4C, SNX16, BC038578, RALYL, LRRCC1, E2F5, AB209185, C8orf59, CA13, CA1, CA3, CA2, FW340046, REXO1L2P, REXO1L1, REXO1L1, PSKH2, ATP6V0D2, SLC7A13, BC038731, Mir_147, WWP1, RMDN1, CPNE3, CNGB3, CNBD1, DCAF4L2, DKFZp761D112, MMP16, RIPK2, OSGIN2, NBN, DECR1, CALB1, LINC00534, LINC01030, BC040572, TMEM64, NECAB1, C8orf88,	0

8	97,721,204	133,066,762	High Copy Gain	35345559	q22.1 - q24.22	CPQ, TSPYL5, MTDH, LAPTM4B, Mir_684, MATN2, RPL30, SNORA72, C8orf47, ERICH5, HRSP12, POP1, TRNA_Und, NIPAL2, KCNS2, STK3, OSR2, VPS13B, MIR599, MIR875, DL491896, COX6C, SNORD77, RGS22, FBXO43, POLR2K, SPAG1, RNF19A, MIR4471, ANKRD46, SNX31, MIR7705, PABPC1, AX746867, YWHAZ, FLJ42969, ZNF706, AK291701, NACAP1, GRHL2, NCALD, MIR5680, RRM2B, AK095151, UBR5, ODF1, KLF10, AK127183, AZIN1, ATP6V1C1, AK001351, BAALCOS, C8orf56, MIR3151, BAALC-AS1, BAALC, LOC100499183, FZD6, CTHRC1, SLC25A32, DCAF13, RIMS2, DCSTAMP, DPYS, LRP12, Mir_584, ZFPM2, OXR1, ABRA, Mir_548, ANGPT1, RSPO2, EIF3E, EMC2, TMEM74, TRHR, NUDCD1, ENY2, PKHD1L1, EBAG9, SNORD112, SYBU, AX748380, U2, KCNV1, MIR2053, CSMD3, TRPS1, LINC00536, EIF3H, UTP23, RAD21, MIR3610, RAD21-AS1, AL832163, AARD, SLC30A8, SNORA31, MED30, EXT1, AK025288, SAMD12,	0

8	133,066,763	146,364,022	CN Gain	13297260	q24.22 - q24.3	OC90, HHLA1, KCNQ3, HPYR1, LRRC6, TMEM71, PHF20L1, TG, TG, MIR7848, SLA, WISP1, AX746885, NDRG1, ST3GAL1, ZFAT, ZFAT-AS1, MIR30B, MIR30D, Mir_652, LOC286094, KHDRBS3, U1, Mir_720, FAM135B, COL22A1, KCNK9, TRAPPC9, AX748239, CHRAC1, DQ574852, AGO2, Mir_28, PTK2, DENND3, SLC45A4, LINC01300, LOC731779, GPR20, AK311257, PTP4A3, MROH5, MIR4472-1, LINC00051, TSNARE1, BAI1, ARC, JH8, JRK, PSCA, AX747544, LY6K, LOC100288181, THEM6, SLURP1, LYPD2, LYNX1, LY6D, GML, CYP11B1, CYP11B2, CDC42P3, LOC100133669, LY6E, C8orf31, LY6H, GPIHBP1, ZFP41, GLI4, ZNF696, TOP1MT, RHPN1-AS1, RHPN1, MAFA, ZC3H3, GSDMD, MROH6, NAPRT1, EEF1D, BC034020, TIGD5, PYCRL, TSTA3, ZNF623, BREA2, ZNF707, CCDC166, MAPK15, FAM83H, MIR4664, FAM83H-AS1, LOC100128338, AX746851, SCRIB, MIR937, PUF60, MIR6845, NRBP2, EPPK1, MIR661, PLEC, PARP10,	0

9	64,626	10,139,240	CN Loss	10074615	p24.3 - p23	AY343892, AY343902, FOXD4, CBWD1, C9orf66, DOCK8, KANK1, DMRT1, DMRT3, U6, LINC01230, DMRT2, SMARCA2, FLJ35024, VLDLR-AS1, VLDLR, KCNV2, KIAA0020, Mir_548, RFX3, BC069756, GLIS3-AS1, GLIS3, Mir_320, SLC1A1, SPATA6L, PPAPDC2, CDC37L1, AK3, RCL1, MIR101-2, AK021739, JAK2, TRNA_Gln, INSL6, INSL4, RLN2, RLN1, CD274, PLGRKT, PDCD1LG2, KIAA1432, ERMP1, MLANA, KIAA2026, MIR4665, RANBP6, IL33, TPD52L3, UHRF2, GLDC, KDM4C, DQ580140, KDM4C, Mir_584, C9orf123, TMEM261, AK094342, SNORD27, Metazoa_SRP, PTPRD	JAK2_pK191Q_c571A_C, JAK2_pY570Y_c1710C_T, JAK2_pV617F_c1849G_T, JAK2_pR683G_c2047A_G
9	10,139,240	10,320,113	Homozygous Copy Loss	180874	p23	PTPRD	0

9	10,320,113	26,205,565	CN Loss	15885453	p23 - p21.2	PTPRD, PTPRD-AS2, JB175300, TYRP1, LURAP1L, MPDZ, FLJ41200, LINC00583, NFIB, TRNA_His, TRNA, ZDHHC21, CER1, FREM1, LOC389705, AK127963, AK021570, TTC39B, SNAPC3, PSIP1, CCDC171, BC042022, BX648501, C9orf92, BNC2, CNTLN, SH3GL2, MIR3152, 7SK, ADAMTSL1, DKFZp686L03130, FAM154A, RRAGA, SCARNA8, HAUS6, PLIN2, AK094196, DQ572382, DENND4C, RPS6, TRNA_Met, ACER2, SLC24A2, MIR4473, U4, MLLT3, MIR4474, MIR491, SNORA30, FOCAD, PTPLAD2, IFNB1, IFNW1, IFNA21, IFNA4, IFNA7, IFNA10, IFNA16, IFNA14, IFNA17, IFNA22P, IFNA5, DKFZp686L0695, KLHL9, IFNA6, IFNA13, IFNA2, IFNA8, IFNA1, IFNE, MIR31HG, MIR31, MTAP, C9orf53, CDKN2A, CDKN2B, Mir_384, CDKN2B-AS1, CDKN2B_AS, DMRTA1, FLJ35282, LINC01239, CR627240, ELAVL2, Hel-N1, IZUMO3, JA375049, JA374875, TUSC1, BC043546, LOC100506422	CDKN2A_p_c457_plus_2T_C, CDKN2A_p_c457_plus_1G_T, CDKN2A_pR131H_c392G_A, CDKN2A_p_c150_plus_2T_C, CDKN2A_pA30V_c89C_T, CDKN2A_pE26X_c76G_T, CDKN2A_pW15X_c44G_A, CDKN2A_p_c1_minus_25C_T, CDKN2A_pE120K_c358G_A, CDKN2A_pW110X_c330G_A, CDKN2A_pW110X_c329G_A, CDKN2A_pD108N_c322G_A, CDKN2A_pE88E_c264G_A, CDKN2A_pH83Y_c247C_T, CDKN2A_pR80X_c238C_T, CDKN2A_pE69X_c205G_T, CDKN2A_pE61X_c181G_T, CDKN2A_pR58X_c172C_T, CDKN2A_p_c151_minus_1G_A, CDKN2A_pP48L_c143C_T, CDKN2A_pY44X_c132C_A, CDKN2A_pS43I_c128G_T, CDKN2A_pG35E_c104G_A
9	26,205,565	27,146,969	CN Gain	941405	p21.2	DQ598339, DQ591605, DQ574229, DQ599043, DQ586829, DQ571132, DQ582785, CAAP1, PLAA, LRRC19, IFT74, TEK	0
9	27,146,970	27,289,413	High Copy Gain	142444	p21.2	TEK, 5S_rRNA, LINC00032, EQTN	0

9	73,037,883	112,950,586	CN Gain	39912704	q21.12 - q31.3	MIR204, TRPM3, TMEM2, ABHD17B, C9orf85, C9orf57, GDA, 5S_rRNA, AK095210, LOC100507540, ZFAND5, TMC1, ALDH1A1, ANXA1, MIR6130, RORB, TRPM6, TRNA_Asp, C9orf40, BC043649, C9orf41, NMRK1, OSTF1, MIR548H3, PCSK5, RFK, RPSAP9, GCNT1, PRUNE2, PCA3, PCA3_1, FOXB2, VPS13A-AS1, VPS13A, GNA14, GNAQ, CEP78, PSAT1, LOC101927450, TLE4, DQ575560, DQ583878, DQ589820, DQ580124, DQ595182, DQ596925, DQ597713, DQ586158, DQ588659, DQ599976, DQ574305, DQ600106, DQ574306, DQ599155, DQ574330, DQ579956, DQ594965, DQ596802, DQ574826, DQ592136, DQ577348, DQ593172, DQ592375, DQ570523, DQ573195, DQ591664, TLE1, LOC101927502, BC036431, SPATA31D5P, DQ592725, DQ577940, DQ578305, DQ582032, DQ584769, SPATA31D4, SPATA31D3, AK097447, SPATA31D1, DQ588544, Mir_1302, RASEF, FRMD3, IDNK, UBQLN1, AK300656, GKAP1, KIF27, C9orf64, MIR7-1, HNRNPK, RMI1,	PTCH1_pW1018X_c3054G_A, PTCH1_pM561R_c1682T_G, PTCH1_pQ417X_c1249C_T, PTCH1_pQ365X_c1093C_T

9	134,275,097	141,213,431	CN Gain	6938335	q34.13 - q34.3	PRRC2B, PRRC2B, SNORD62A, SNORD62A, SNORD62B, LQFBS-1, POMT1, UCK1, RAPGEF1, DD413682, MED27, NTNG2, SETX, TTF1, C9orf171, BARHL1, DDX31, GTF3C4, AK8, AX748058, C9orf9, TSC1, AJ011378, GFI1B, GTF3C5, MIR6877, CEL, CELP, KIAA1308, RALGDS, GBGT1, OBP2B, ABO, SURF6, MED22, SNORD24, RPL7A, SNORD36B, SNORD36A, DQ601906, SNORD36C, SURF1, SURF2, SURF4, C9orf96, REXO4, ADAMTS13, CACFD1, SLC2A6, TMEM8C, ADAMTSL2, FAM163B, DBH, DBH-AS1, SARDH, AK123314, VAV2, LINC00094, BRD3, WDR5, RNU6ATAC, RXRA, MIR4669, COL5A1, LOC101448202, MIR3689C, MIR3689D1, MIR3689A, MIR3689B, MIR3689D2, MIR3689E, MIR3689F, FCN2, FCN1, OLFM1, LOC401557, C9orf62, LOC100506599, PPP1R26-AS1, AK096249, PPP1R26, C9orf116, MRPS2, BC015688, LCN1, OBP2A, PAEP, LOC100130954, GLT6D1, LCN9, BC101937, SOHLH1, KCNT1, CAMSAP1, UBAC1, NACC2, C9orf69,	NOTCH1_pQ2460X_c7378C_T, NOTCH1_pL1679P_c5036T_C, NOTCH1_pL1601P_c4802T_C, NOTCH1_pL1594P_c4781T_C, NOTCH1_pL1586P_c4757T_C, NOTCH1_pL1575P_c4724T_C
10	0	712,669	CN Loss	712670	p15.3	TUBB8, ZMYND11, 5S_rRNA, DIP2C, DIP2C, MIR5699, PRR26	0
10	49,837,396	49,854,701	CN Loss	17306	q11.22	ARHGAP22	0

10	60,551,322	67,751,830	CN Gain	7200509	q21.1 - q21.3	BICC1, LINC00844, PHYHIPL, Mir_584, FAM13C, SLC16A9, LINC00948, CCDC6, C10orf40, Metazoa_SRP, ANK3, CDK1, U2, RHOBTB1, LINC00845, TMEM26, BC041470, C10orf107, MIR548AV, ARID5B, RTKN2, LOC283045, ZNF365, ADO, EGR2, NRBF2, JMJD1C, AX747628, JMJD1C, MIR1296, JMJD1C-AS1, REEP3, DJ439558, ANXA2P3, DJ439576, DJ439561, CTNNA3	0
10	67,751,831	67,792,448	CN Loss	40618	q21.3	CTNNA3	0

10	85,557,432	105,804,295	CN Loss	20246864	q23.1 - q25.1	GHITM, BC051760, C10orf99, CDHR1, LRIT2, LRIT1, LRRC21, RGR, LINC00858, DD413707, CCSER2, Mir_544, AK097624, GRID1-AS1, AX746544, GRID1, MIR346, WAPAL, WAPL, OPN4, LDB3, AX747977, BMPR1A, MMRN2, SNCG, AL157440, ADIRF, AGAP11, FAM25A, GLUD1, FAM35A, NUTM2A, NUTM2A-AS1, LOC439994, BC082979, NUTM2D, LINC00864, MIR4678, MINPP1, PAPSS2, ATAD1, CFL1P1, KLLN, PTEN, AK130076, RNLS, LIPJ, LIPF, LIPK, LIPN, LIPM, ANKRD22, BC069782, STAMBPL1, AX748062, ACTA2, FAS-AS1, FAS, HV303528, HV303525, MIR4679-2, MIR4679-1, CH25H, IFIT2, BC040833, LIPA, IFIT3, IFIT1B, IFIT1, IFIT5, SLC16A12, JB022994, MIR107, PANK1, FLJ37201, KRMP1, KIF20B, LINC00865, LOC101926924, AK093219, LOC101926942, BC037970, HTR7, Y_RNA, RPP30, ANKRD1, LINC00502, NUDT9P1, PCGF5, LOC100188947, HECTD2, PPP1R3C, TNKS2-AS1, TNKS2, FGFBP3, BTAF1, SNORA25, CPEB3,	PTEN_pE7X_c19G_T, PTEN_pR15I_c44G_T, PTEN_pQ17X_c49C_T, PTEN_pL23F_c69A_C, PTEN_pP38S_c112C_T, PTEN_pL42R_c125T_G, PTEN_p_c165_minus_2A_C, PTEN_pH61R_c182A_G, PTEN_pY68H_c202T_C, PTEN_p_c209_plus_5G_A, PTEN_p_c253_plus_1G_A, PTEN_p_c253_plus_1G_T, PTEN_pH93Y_c277C_T, PTEN_pH93Q_c279T_G, PTEN_pP95L_c284C_T, PTEN_pI101T_c302T_C, PTEN_pD107Y_c319G_T, PTEN_pQ110X_c328C_T, PTEN_pA126T_c376G_A, PTEN_pG129R_c385G_A, PTEN_pR130X_c388C_T, PTEN_pR130Q_c389G_A, PTEN_pA151T_c451G_A, PTEN_pY155C_c464A_G, PTEN_pG165R_c493G_A, PTEN_pQ171X_c511C_T, PTEN_pR173C_c517C_T,

11	0	11,638,440	CN Loss	11638441	p15.5 - p15.3	LINC01001, AL137655, LOC100133161, LOC653486, SCGB1C1, ODF3, BET1L, MIR6743, RIC8A, Z49985, SIRT3, PSMD13, NLRP6, ATHL1, IFITM5, IFITM2, IFITM1, IFITM3, BC040735, B4GALNT4, PKP3, SIGIRR, ANO9, Metazoa_SRP, PTDSS2, RNH1, HRAS, LRRC56, C11orf35, AX748330, BC031953, RASSF7, MIR210HG, MIR210, LOC143666, PHRF1, IRF7, CDHR5, SCT, DRD4, DEAF1, TMEM80, EPS8L2, TALDO1, BC048998, PDDC1, NS3BP, CEND1, SLC25A22, PIDD, PIDD1, RPLP2, SNORA52, PNPLA2, EFCAB4A, JB050151, CD151, POLR2L, TSPAN4, AK126635, AX747537, CHID1, AP2A2, MUC6, MUC2, MUC5B, MUC5AC, MIR6744, TOLLIP, TOLLIP-AS1, BRSK2, MOB2, DUSP8, KRTAP5-1, KRTAP5-AS1, KRTAP5-2, KRTAP5-3, KRTAP5-4, KRTAP5-5, FAM99A, FAM99B, AF085962, KRTAP5-6, IFITM10, CTSD, SYT8, TNNI2, MIR4298, LSP1, TNNT3, MRPL23, AK126380, MRPL23-AS1, AK311497, H19, MIR675, MIR483, IGF2, IGF2-AS,	HRAS_pQ61H_c183G_T, HRAS_pQ61P_c182A_C, HRAS_pQ61K_c181C_A, HRAS_pG13D_c38G_A, HRAS_pG13S_c37G_A, HRAS_pG12D_c35G_A, HRAS_pG12S_c34G_A
11	11,638,440	13,150,261	CN Gain	1511822	p15.3 - p15.2	GALNT18, MIR4299, MIR8070, USP47, DKK3, MIR6124, MICAL2, AK026905, MICALCL, PARVA, DD413619, TEAD1, SCARNA16, LINC00958, LOC100506305, RASSF10	0

11	13,150,261	13,436,450	High Copy Gain	286190	p15.2	ARNTL, 7SK, BTBD10	0
11	13,436,450	22,045,341	CN Gain	8608892	p15.2 - p14.3	BTBD10, PTH, FAR1, SPON1, RRAS2, COPB1, U7, PSMA1, PDE3B, CYP2R1, CALCA, CALCB, INSC, SnoMBII_202, MIR6073, SOX6, Metazoa_SRP, C11orf58, AL833346, PLEKHA7, OR7E14P, SNORD14, RPS13, PIK3C2A, NUCB2, AK096475, NCR3LG1, KCNJ11, ABCC8, USH1C, OTOG, MYOD1, KCNC1, SERGEF, TPH1, SAAL1, SAA3P, MRGPRX3, MRGPRX4, LOC494141, SAA4, SAA2-SAA4, SAA2, SAA1, HPS5, GTF2H1, LDHA, LDHC, LDHAL6A, TSG101, JA429845, UEVLD, SPTY2D1-AS1, SPTY2D1, TMEM86A, IGSF22, PTPN5, MRGPRX1, MRGPRX2, ZDHHC13, Mir_340, CSRP3, E2F8, NAV2-AS5, NAV2-AS4, MIR4486, SNORA1, LOC100126784, NAV2, MIR4694, DBX1, TRNA, HTATIP2, PRMT3, SLC6A5, NELL1	0
11	26,084,277	26,110,050	CN Loss	25774	p14.3 - p14.2	0	0
11	27,682,693	28,114,125	CN Gain	431433	p14.1	BDNF-AS, BDNF, MIR610, JB074938, KIF18A	0
11	28,114,126	28,452,521	High Copy Gain	338396	p14.1	KIF18A, METTL15	0
11	28,452,522	28,566,789	CN Gain	114268	p14.1	MIR8068	0
11	28,566,790	29,034,634	High Copy Gain	467845	p14.1	0	0

11	29,062,789	29,110,258	High Copy Gain	47470	p14.1	0	0
11	29,272,732	34,694,617	CN Gain	5421886	p14.1 - p13	KCNA4, FSHB, ARL14EP, Mir_633, MPPED2, DCDC5, DCDC1, DNAJC24, IMMP1L, ELP4, PAX6, PAUPAR, DKFZp686K1684, RCN1, U3, WT1, WT1-AS, WT1, EIF3M, CCDC73, PRRG4, QSER1, DEPDC7, TCP11L1, LINC00294, CSTF3, CSTF3-AS1, HIPK3, KIAA1549L, C11orf91, CD59, FBXO3, LMO2, CAPRIN1, NAT10, ABTB2, CAT, ELF5, EHF	WT1_pR394W_c1180C_T, WT1_pR390X_c1168C_T, WT1_pG379V_c1136G_T, WT1_pR362X_c1084C_T, WT1_pS313X_c938C_A, WT1_pR301X_c901C_T, WT1_pF154S_c461T_C, WT1_pS46X_c137C_A
11	34,752,447	37,597,733	CN Gain	2845287	p13 - p12	APIP, MIR1343, PDHX, CD44, SLC1A2, PAMR1, FJX1, TRIM44, DQ573949, Mir_652, MIR3973, BC036209, LDLRAD3, COMMD9, PRR5L, TRAF6, RAG1, RAG2, C11orf74	0

11	71,188,902	73,965,549	CN Loss	2776648	q13.4	NADSYN1, KRTAP5-7, KRTAP5-8, KRTAP5-9, KRTAP5-10, KRTAP5-11, FAM86C1, ALG1L9P, ZNF705E, DEFB108B, LOC100129216, LOC100133315, RNF121, IL18BP, LOC100128494, NUMA1, MIR3165, LRTOMT, LAMTOR1, ANAPC15, FOLR3, FOLR1, FOLR2, INPPL1, PHOX2A, CLPB, AL832797, MIR139, PDE2A, BC150585, ARAP1, STARD10, MIR4692, ATG16L2, FCHSD2, P2RY2, P2RY6, ARHGEF17, RELT, FAM168A, PLEKHB1, RAB6A, MRPL48, COA4, PAAF1, DNAJB13, UCP2, UCP3, C2CD3, PPME1, SNORA7, P4HA3	0
12	169,821	1,143,708	CN Gain	973888	p13.33	IQSEC3, LOC574538, SLC6A12, LOC101929384, SLC6A13, LOC102723544, KDM5A, CCDC77, B4GALNT3, NINJ2, LOC100049716, U4atac, WNK1, HSN2, AK128619, RAD52, BC039168, ERC1	0
12	1,231,447	1,654,094	CN Gain	422648	p13.33	ERC1, LINC00942, AX746535	0
12	2,160,748	3,577,145	CN Gain	1416398	p13.33 - p13.32	AK308652, CACNA1C-AS4, CACNA1C-IT3, CACNA1C, Mir_584, CACNA1C-AS2, CACNA1C-AS1, LOC283440, FKBP4, ITFG2, NRIP2, LOC100507424, FOXM1, RHNO1, TULP3, TEAD4, TSPAN9, AK056228, AK095365, BC047090, DQ574680, DQ597527, DQ591569, DQ590472, PRMT8	0

12	5,314,214	6,032,797	CN Loss	718584	p13.32 - p13.31	LOC101929584, BC031884, NTF3, ANO2	0
12	6,426,433	7,806,728	CN Loss	1380296	p13.31	PLEKHG6, TNFRSF1A, SCNN1A, LTBR, CD27-AS1, CD27, TAPBPL, VAMP1, MRPL51, SCARNA10, NCAPD2, GAPDH, DL491527, IFFO1, NOP2, SCARNA11, AK096395, CHD4, LPAR5, ACRBP, ING4, ZNF384, PIANP, COPS7A, MLF2, PTMS, LAG3, CD4, GPR162, LEPREL2, GNB3, AK097957, CDCA3, USP5, TPI1, SPSB2, RRPL13L, RPL13P5, DSTNP2, LRRC23, ENO2, ATN1, C12orf57, PTPN6, MIR200C, MIR141, SCARNA12, PHB2, EMG1, LPCAT3, C1S, C1R, C1RL, C1RL-AS1, RBP5, CLSTN3, PEX5, ACSM4, CD163L1, CD163, APOBEC1	0
12	7,806,728	8,485,813	CN Gain	679086	p13.31	APOBEC1, GDF3, DPPA3, CLEC4C, NANOGNB, NANOG, Y_RNA, SLC2A14, NANOGP1, AY455283, SLC2A3, FOXJ2, C3AR1, BC039122, NECAP1, CLEC4A, POU5F1P3, ZNF705A, FAM66C, FAM90A1, FAM86FP, LOC101927905	0

12	8,485,813	32,425,114	CN Loss	23939302	p13.31 - p11.21	LINC00937, CLEC6A, CLEC4D, CLEC4E, AICDA, MFAP5, RIMKLB, A2ML1, PHC1, M6PR, KLRG1, LINC00612, A2M-AS1, A2M, JB175316, PZP, A2MP1, MIR1244-1, MIR1244-2, MIR1244-3, LINC00987, LOC100499405, SNORA75, LOC642846, DQ582774, DQ586985, DQ577612, DQ586485, DQ577180, DQ585706, DQ575612, DQ592342, LOC101928030, LOC101930452, DQ599803, DDX12P, SNORA75, BX647938, KLRB1, LOC374443, CLEC2D, CLECL1, CD69, KLRF1, CLEC2B, KLRF2, CLEC2A, LOC100506159, CLEC12A, CLEC1B, CLEC12B, LOC102724020, CLEC9A, CLEC1A, CLEC7A, OLR1, BC055085, TMEM52B, GABARAPL1, KLRD1, LOC101928100, AK096314, KLRK1, KLRC4-KLRK1, KLRC4, KLRC3, KLRC2, KLRC1, KLRAP1, MAGOHB, STYK1, YBX3, LOC101928162, TAS2R7, TAS2R8, TAS2R9, TAS2R10, PRR4, TAS2R13, PRH2, TAS2R14, TAS2R50, TAS2R20, PRH1-PRR4, TAS2R19, PRH1, TAS2R31, TAS2R46, TAS2R43, PRB4, TAS2R30, LOC100129361,	KRAS_pA146T_c436G_A, KRAS_pQ61H_c183A_C, KRAS_pQ61P_c182A_C, KRAS_pQ61K_c181C_A, KRAS_pA59T_c175G_A, KRAS_pG13D_c38G_A, KRAS_pG13S_c37G_A, KRAS_pG12D_c35G_A, KRAS_pG12S_c34G_A
12	32,996,159	34,244,646	CN Loss	1248488	p11.21 - p11.1	PKP2, SYT10, ALG10	0
12	72,458,905	75,122,435	CN Loss	2663531	q21.1	TRHDE-AS1, BC093903, TRHDE, LOC101928137, LOC100507377, TRNA_Gln, ATXN7L3B	0

13	19,020,013	32,178,877	CN Loss	13158865	q11 - q12.3	LINC00417, DQ586768, DQ579288, DQ587539, ANKRD20A9P, BC035261, LINC00408, DQ572285, LINC00442, RNU6-52P, TUBA3C, ANKRD26P3, LINC00421, TPTE2, MPHOSPH8, PSPC1, ZMYM5, BC044596, ZMYM2, GJA3, GJB2, GJB6, MIR4499, CRYL1, IFT88, IL17D, AK055408, N6AMT2, XPO4, LINC00367, LATS2, SAP18, SKA3, MRPL57, MRP63, LINC00539, MIPEPP3, ZDHHC20, MICU2, FGF9, Metazoa_SRP, LINC00424, AK054845, BC035104, LINC00540, BC048997, BASP1P1, SGCG, SACS, SACS-AS1, LINC00327, TNFRSF19, MIPEP, C1QTNF9B-AS1, C1QTNF9B, ANKRD20A19P, BC043582, SPATA13, MIR2276, SPATA13-AS1, C1QTNF9, BC038727, PARP4, TPTE2P6, ATP12A, RNF17, CENPJ, AK054988, TPTE2P1, PABPC3, AMER2, LOC101928922, BC022569, LOC102723318, MTMR6, NUPL1, ATP8A2, SHISA2, RNF6, CDK8, BC047364, WASF3, GPR12, USP12, USP12-AS1, USP12-AS2, LINC00412, RPL21P28, RPL21, SNORD102, SNORA27, RASL11A, GTF3A, MTIF3,	FLT3_pD835E_c2505T_G, FLT3_pD835V_c2504A_T_allele1, FLT3_pD835N_c2503G_A, FLT3_pL561L_c1683A_G

13	32,178,877	33,860,144	Homozygous Copy Loss	1681268	q12.3 - q13.1	RXFP2, EEF1DP3, DKFZp666K117, BC035084, FRY-AS1, FRY, ZAR1L, BRCA2, N4BP2L1, SNORA16, HV983084, N4BP2L2, MINOS1P1, N4BP2L2-IT2, U50535, BC026240, PDS5B, LINC00423, KL, STARD13-AS, STARD13	BRCA2_pR18H_c53G_A,<br>BRCA2_pE187K_c559G_A,<br>BRCA2_pT630I_c1889C_T,<br>BRCA2_pP920S_c2758C_T,<br>BRCA2_pI1017S_c3050T_G,<br>BRCA2_pG1338G_c4014C_T,<br>BRCA2_pE1593X_c4777G_T,<br>BRCA2_pS1682S_c5046T_C,<br>BRCA2_pV1988I_c5962G_A,<br>BRCA2_pH2415N_c7243C_A,<br>BRCA2_pR2678S_c8034G_T,<br>BRCA2_pR2787H_c8360G_A,<br>BRCA2_pR2842C_c8524C_T,<br>BRCA2_pQ2934X_c8800C_T,<br>BRCA2_pD3095E_c9285C_A,<br>BRCA2_pI3103M_c9309A_G,<br>BRCA2_pR3128X_c9382C_T

13	33,860,144	109,025,409	CN Loss - includes *RB1*	75165266	q13.1 - q33.3	STARD13, U5, RFC3, LINC00457, NBEA, MAB21L1, MIR548F5, DCLK1, TRNA_Pseudo, SOHLH2, CCDC169-SOHLH2, CCDC169, U6, SPG20, SPG20OS, CCNA1, SERTM1, 5S_rRNA, RFXAP, SMAD9, ALG5, EXOSC8, SUPT20H, CSNK1A1L, LINC00547, POSTN, TRPC4, Mir_720, LINC00571, UFM1, LINC00366, FREM2, STOML3, PROSER1, NHLRC3, LHFP, AK021977, MIR4305, COG6, LINC00332, LINC00548, 7SK, LINC00598, FOXO1, MIR320D1, MRPS31, SLC25A15, MIR621, TPTE2P5, SUGT1P3, ELF1, TRNA_Glu, WBP4, KBTBD6, LOC101929140, AK056182, KBTBD7, BC051736, MTRF1, Mir_324, NAA16, U6, OR7E37P, RGCC, MIR5006, VWA8, Mir_684, VWA8-AS1, DGKH, AK054970, AKAP11, TNFSF11, FAM216B, EPSTI1, DNAJC15, LINC00400, ENOX1-AS2, ENOX1, CCDC122, LACC1, LINC00284, SMIM2-AS1, SMIM2, MGC5590, SMIM2-IT1, MIR8079, AX748251, SERP2, BC025370, TUSC8, TSC22D1, TSC22D1-AS1, LINC00330, TRNA, NUFIP1,	RB1_pE54X_c160G_T, RB1_pS82X_c245C_A, RB1_p_c380_plus_1G_A, RB1_pE137X_c409G_T, RB1_pQ217X_c649C_T, RB1_pR251X_c751C_T, RB1_pR320X_c958C_T, RB1_pR358X_c1072C_T, RB1_pQ395X_c1183C_T, RB1_p_c1215_plus_1G_A, RB1_pE440X_c1318G_T, RB1_pR445X_c1333C_T, RB1_pR455X_c1363C_T, RB1_p_c1499_minus_1G_T, RB1_pR552X_c1654C_T, RB1_pR556X_c1666C_T, RB1_pR579X_c1735C_T, RB1_p_c1961_minus_1G_A, RB1_pQ685X_c2053C_T, RB1_pQ702X_c2104C_T, RB1_p_c2107_minus_2A_G, RB1_pE748X_c2242G_T, RB1_p_c2326_minus_2A_C, RB1_pR787X_c2359C_T

13	111,794,504	113,487,192	CN Loss	1692689	q34	ARHGEF7-AS1, LOC101060553, ARHGEF7, AX748212, TEX29, LINC00354, LINC00403, SOX1, AK055145, LINC01070, CR627049, LOC101928730, SPACA7, TUBGCP3, C13orf35, ATP11AUN, BC041346, ATP11A-AS1, AX748036, ATP11A		0
16	34,196,182	34,920,840	CN Loss	724659	p11.2 - p11.1	UBE2MP1, LOC283914, LOC146481, LOC100130700		0

16	46,455,960	90,354,753	CN Loss	43898794	q11.2 - q24.3	ANKRD26P1, SHCBP1, VPS35, ORC6, MYLK3, C16orf87, GPT2, DNAJA2, NETO2, LOC101927102, ITFG1, Y_RNA, PHKB, BC048130, LOC100507534, LOC101927132, ABCC12, ABCC11, LONP2, MIR548AE2, LOC100507577, SIAH1, N4BP1, U6, CBLN1, C16orf78, ZNF423, CNEP1R1, HEATR3, AF086132, PAPD5, ADCY7, MIR6771, BRD7, SNORD112, NKD1, LOC101927272, AF143871, SNX20, NOD2, CYLD, LOC101927334, SALL1, LOC101927364, C16orf97, LINC00919, LOC102467079, TOX3, LINC00918, CASC16, CHD9, LOC643802, LOC102723373, RBL2, AKTIP, RPGRIP1L, FTO, JB149426, FTO-IT1, IRX3, CRNDE, IRX5, IRX6, MMP2, LPCAT2, CAPNS2, SLC6A2, CES1P2, CES1P1, CES1, CES5A, LOC283856, DKFZP434H168, MIR3935, GNAO1, AMFR, NUDT21, OGFOD1, BBS2, MT4, MT3, MT2A, MT1L, MT1A, MT1M, MT1E, MT1JP, MT1DP, MT1B, MT1F, MT1G, MT1H, MTE, MT1IP, MT1X, NUP93, MIR138-2, SLC12A3, MIR6863, HERPUD1, CETP, NLRC5,	CDH1_p_c1009_minus_1G_A, CDH1_pI374I_c1122C_T

17	7,506,837	7,671,804	CN Loss includes *TP53*	164968	p13.1	FXR2, TRNA_Pseudo, SHBG, SAT2, ATP1B2, HV941431, HV941433, HV941428, HV941434, HV941486, HV941429, TP53, HV941440, HV941478, HV941442, HV941444, HV941430, WRAP53, EFNB3, RPL29P2, DNAH2	TP53_pR342X_c1024C_T, TP53_pE336X_c1006G_T, TP53_pQ331X_c991C_T, TP53_p_c920_minus_1G_A, TP53_pR306X_c916C_T, TP53_pE298X_c892G_T, TP53_pE285K_c853G_A, TP53_pR273H_c818G_A, TP53_pR273C_c817C_T, TP53_pG266E_c797G_A, TP53_p_c782_plus_1G_T, TP53_pR249S_c747G_T, TP53_pR248Q_c743G_A, TP53_pR248W_c742C_T, TP53_pG245S_c733G_A, TP53_pY236C_c707A_G, TP53_p_c672_plus_1G_A, TP53_pY220C_c659A_G, TP53_pR213X_c637C_T, TP53_pR196X_c586C_T, TP53_pH193R_c578A_G, TP53_pH179Q_c537T_G, TP53_pH179R_c536A_G, TP53_pC176F_c527G_T, TP53_pR175H_c524G_A, TP53_pY163C_c488A_G, TP53_pA159V_c476C_T,
17	7,728,166	7,760,539	CN Loss	32374	p13.1	DNAH2, KDM6B, TMEM88, LSMD1, NAA38	0
17	60,940,628	61,006,083	CN Loss	65456	q23.2	MIR548W	0
17	61,144,186	61,439,735	CN Loss	295550	q23.3	MIR548W, TANC2	0
17	61,454,707	61,650,448	CN Loss	195742	q23.3	TANC2, CYB561, ACE, KCNH6, DCAF7	0

18	41,334,022	41,339,844	CN Gain	5823	q12.3	0	0
19	6,531,407	6,609,287	CN Loss	77881	p13.3	TNFSF9, CD70	0
20	0	14,782,559	CN Loss	14782560	p13 - p12.1	DEFB125, DEFB126, DEFB127, DEFB128, DEFB129, DEFB132, C20orf96, ZCCHC3, LOC100507459, SOX12, NRSN2, TRIB3, RBCK1, DQ588114, TBC1D20, CSNK2A1, TCF15, SRXN1, SCRT2, SLC52A3, FAM110A, ANGPT4, RSPO4, PSMF1, TMEM74B, C20orf202, RAD21L1, SNPH, SDCBP2, FKBP1A-SDCBP2, SDCBP2-AS1, FKBP1A, MIR6869, NSFL1C, SIRPB2, SIRPD, SIRPB1, AK093519, LOC101929010, SIRPG, LOC100289473, SIRPA, AK090681, PDYN, STK35, LOC388780, TGM3, TGM6, SNORD119, SNRPB, AK126717, ZNF343, TMC2, MIR1292, SNORD110, SNORA51, NOP56, SNORD86, SNORD56, SNORD57, IDH3B, EBF4, CPXM1, C20orf141, TMEM239, PCED1A, VPS16, PTPRA, GNRH2, MRPS26, OXT, AVP, UBOX5-AS1, UBOX5, FASTKD5, LZTS3, DDRGK1, ITPA, SLC4A11, C20orf194, U6, ATRN, GFRA4, AX748440, ADAM33, SIGLEC1, HSPA12B, C20orf27, SPEF1, CENPB, CDC25B, LOC101929125, BC042140, AP5S1, MAVS, BC012193, PANK2,	PAK7_pT397K_c1190C_A
20	14,782,560	14,827,300	Homozygous	44741	p12.1	MACROD2, MACROD2	0

20	14,827,301	26,319,438	CN Loss	11492138	p12.1 - p11.1	MACROD2, MACROD2-AS1, MACROD2, AK125594, Mir_584, KIF16B, SNRPB2, OTOR, PCSK2, BFSP1, DSTN, RRBP1, BANF2, TRNA_Gln, SNX5, SNORD17, AK296947, MGME1, JB175279, OVOL2, PET117, CSRP2BP, ZNF133, BC043266, LINC00851, DZANK1, MIR3192, POLR3F, RBBP9, SEC23B, LINC00493, DTD1, LOC101929526, LINC00652, LOC100270804, SCP2D1, C20orf78, LOC100130264, SLC24A3, Mir_548, BC090059, DQ594620, DQ573663, RIN2, NAA20, CRNKL1, C20orf26, INSM1, RALGAPA2, BC042893, PLK1S1, KIZ, LOC101929591, BC034426, XRN2, NKX2-4, NKX2-2, LOC101929625, Nkx2_2as, LOC101929608, PAX1, LOC100270679, CR627206, LOC101929663, LOC284788, LINC00261, FOXA2, LOC101929685, AK055550, BC045663, SSTR4, AX747264, THBD, CD93, LINC00656, AX747171, NXT1, LOC100505683, GZF1, NAPB, CSTL1, CST11, CST8, CST13P, CST9L, CST9, CST3, CST4,	0
X	23,428,703	23,458,054	CN Loss	29352	p22.11	0	0
X	150,801,467	151,067,028	CN Loss	265562	q28	PASD1, PRRG3, FATE1, CNGA2	0
Y	1	59,373,566	CN Loss - includes *PCDH11Y*	59373566	p11.32 - q12	PLCXD1, GTPBP6, LINC00685, PPP2R3B, SHOX, DQ576039, CSF2RA, MIR3690, IL3RA, CRLF2, SLC25A6,	0
